# Physicochemical and sensory quality of yogurt incorporated with pectin from peel of *Citrus sinensis*

**DOI:** 10.1002/fsn3.400

**Published:** 2016-07-27

**Authors:** Fatiha Arioui, Djamel Ait Saada, Abderrahim Cheriguene

**Affiliations:** ^1^ Laboratory of Food Technology and Nutrition (LTAN)Department of AgronomyUniversity Abdelhamid Ibn BadisMostaganem27000Algeria

**Keywords:** Pectin, preservation, quality, texture, yogurt

## Abstract

Industrial by‐product like orange peel plays an important role in pectin manufacture. The objective of this article was to extract pectin from peel of *Citrus sinensis* and to study the effect of its incorporation on the quality of yogurt during the period of fermentation and postacidification. Physicochemical, organoleptic, and rheological properties of yogurt prepared with pectin were studied in order to determine the best preparation depending on the rate of pectin. The extraction pectin yield was estimated to more than 24%. The viscosity and acidity were increased with increasing of the pectin rate. The best viscosity value was obtained with 0.6% of pectin. Furthermore, the effect of the rate of pectin incorporation in the fermented milks was clearly observed on the number of *Streptococcus thermophilus* and *Lactobacillus bulgaricus*, the cohesiveness, the adhesiveness, the taste, and the whey exudation.

## Introduction

Fermented milk is a dairy product processed by lactic fermentation resulting in acidification and a coagulation pH of about 4.6 of milk casein. Yogurt is one of the most popular fermented dairy product widely consumed all over the word because of its organoleptic and nutritional properties are much sought after by consumers (Sahan et al. 2008; Loveday et al. 2013). It is obtained by lactic fermentation of two specific strains: *Streptococcus thermophilus* and *Lactobacillus bulgaricus* (Vignola 2002; Kumar and Mishra 2004; Sokolinska et al. 2004). The finished product set or stirred must include approximately 10^7^ of these live bacteria/mL (Jeantet et al. 2008).

The texture is a fundamental characteristic of yogurt. It can be improved by the use of gelling, thickening, or stabilizing agents Lucey and Singh (1998). Among these additives, water‐soluble macromolecules or hydrocolloid, the pectin is mainly extracted from apple pomace and citrus peel. Pectin is widely used as a functional ingredient in the food industry due to its ability to form aqueous gels and has been used in jams and jellies, fruit preparations, fruit drink concentrates, fruit juice, desserts, and fermented dairy products (Everett and McLeod 2005).

Pectin, a family of complex heteropolysaccharides consisting predominantly of partially methoxylated galacturonic acid residues, is extensively distributed in almost all of the fruits and vegetables as the structural unit of fresh cells and the junction between the cells. Its structure is based on 1, 4‐linked ‐d‐galacturonic acid, interrupted by l‐rhamnose residues with side chains of neutral sugars (mainly d‐galactose and l‐arabinose) (Guo et al. 2012).

To overcome pollution problems and to get more return in the citrus industry, these residues can be utilized in the production of pectin, which has a wide application in the food and pharmaceutical industries. The aim of this study was to extract pectin from orange peel (*Citrus sinensis*) which is a by‐product of the juice industry. The pectin was then incorporated at different levels in order to improve the texture and sensorial characteristics of yogurt during the fermentation as well as storage period.

## Methodology

### Raw material

The raw material used for the extraction of pectin is the orange peel of *Citrus sinensis* L. variety drawn in Chlef region in the north of Algeria on December 2014. The peel was separated from the endocarp. These represent 28% by weight relative to the fruit. These peels were dried at 50°C for 24 h in an oven, and then placed in hermetically sealed bags until their subsequent uses.

### Extraction of pectin

Pectin was extracted according to the method of Rezzoug et al. (2008). Pectin was extracted from orange peel (*Citrus sinensis*) in hot acid solution, followed by precipitation in a solution of ethyl alcohol at 96°. The dried orange peels were milled during 20 sec, the milled product (10 g) was added to 0.1 N HCl solution (200 mL), and boiled in a reflux system at 90°C for 40 min and then plunged in ice to stop the hydrolysis process. The supernatant were recovered after filtration. The pectin was precipitated with two volumes of alcohol 96° for one volume of supernatant. The obtained precipitate was washed with one volume of alcohol 96°. The pellet is collected, dried in an oven at 40°C for 18 h, and was finally ground to a powder. The yield of pectin was expressed in dry extracted material/100 g dry peel.

### Yogurt manufacture

The reconstituted milk used was prepared at a rate of 140 g/L of milk powder (26% fat). The milk was homogenized and heated to 90°C for 3 min for pasteurization. Once cooled to 45°C, the pectin was respectively incorporated into the milk samples at levels of 0, 0.1, 0.3, and 0.6%. Inoculation of specific lactic strains of yogurt (CHR HANSEN Denmark) was carried out a leaven of 3% (3ml of leaven in 300ml of reconstituted milk) and report of strains *Streptococcus thermophilus* (YC‐X16) and *Lactobacillus bulgaricus* (CHN‐11) of 2S/1L. Each experimental parameter was represented in triplicate tests, with three pots of 100 mL. After incubation of the samples at a temperature of 45°C for 4 h of the fermentation phase, yogurt added with pectin were cooled and preserved at 4°C for 21 days postacidification period.

### Measurement and control

The physicochemical and microbiological analyzes were carried out at 2 h and 4 h during the period of fermentation, while during the period of postacidification, they were made weekly for a period of 21 days of storage at 4°C.

#### Physicochemical analysis

The physicochemical analyses were carried out according to AOAC (2005) methods.

##### pH and acidity

The pH measurement was carried out by a pH meter calibrated with two solutions: one basic and one acidic and at a temperature of 25°C.

The Dornic acidity was determined by titration of 10 mL of yogurt with 0.1 N NaOH using phenolphthalein as an indicator color. Results were expressed as degree Dornic (AFNOR, 1980).

##### Viscosity

Viscosity was measured using a falling ball viscometer using a glass tube and a normalized ball equipped with a chronometer at 25°C. The viscosity was expressed in Pascal sec (Pas).

#### Microbiological analysis

The enumeration of *Streptococcus thermophilus* and *Lactobacillus bulgaricus* was carried out according to the method described by the International Dairy Federation (IDF Standard 117) (International Dairy Federation, 2003). M17 agar was used for the enumeration of *Streptococcus thermophilus* and MRS agar was used for the enumeration of *Lactobacillus bulgaricus*. Microbiological count data are expressed as colony‐forming units (CFU) per mL of yogurt.

#### Organoleptic test

Throughout the period of postacidification (7th, 14th, and 21st days of storage at 4°C), the organoleptic quality of experimental yogurt will be evaluated by a jury of panelists with 10‐point scale. The organoleptic test consists in appreciating the experimental yogurt according to five parameters: taste, cohesiveness, adhesiveness, aftertaste, and whey exudation.

### Statistical treatment

The results of the physicochemical and microbiological analyses were statistically treated by a factorial monovariance analysis in total randomization, followed by comparison of means tow a tow according to the test of Newman and Keuls. However, those relating to the organoleptic test were treated according to the nonparametric test of Friedman (Stat Box 6.4).

## Results and Discussion

### Pectin yield

The extraction yield of pectin was 24.33%. Maran et al. (2013) found that the maximum of yield pectin from orange albedo approximated a rate of 19.24%, while Zanella and Taranto (2015) found that the best yield pectin from albedo of orange (*Citrus sinensis* L. Osbeck) is 38.21%. The results obtained by Guo et al. (2012) showed that this yield was of the order of 15.47%. This value varies according to the extraction parameters (pH, time, and temperature) as well as the characteristics of the raw material (Fishman et al. 2000). Kalapathy and Proctor (2001) showed that a lower temperature and shorter extraction time lead to a lower yield of pectin extraction. Acid strength used for extracting pectin from citrus peel, and the nature of alcohol used during pectin precipitation, had significant effect on the pectin yield extraction.

The heating of the solution of HCl allows the hydrolysis of pectin and other pectic components held in the cell wall (protopectin), thereby increasing the yield of pectin. Chan and Choo (2013) found that low temperature may be insufficient for the hydrolysis of protopectin (the insoluble form of pectin) by acids, which leads to a lower yield of pectin.

[Correction added on 21 October 2016, after first online publication: Reference “Zanelle and Taranto (2015) ” has been changed to “Zanella and Taranto (2015)”.]

### Acidity and pH

The results of the acidity and pH evaluation of yogurt supplemented with pectin were presented in Table 1.

**Table 1:** Evolution of pH and lactic acidity (D°) of yogurt added with pectin

Periods		Measures	Pectin added doses (%)				Effect of pectin incorporation
			0%	0.1%	0.3%	0.6%	
Fermentation	2h	pH	5.34^a ^± 0.01	5.18^b ^± 0.07	4.92^c ^± 0.02	4.92^c ^± 0.03	a
		Acidity D	68.33^b ^± 11.24	54.33^c ^± 3.21	70.5^b ^± 6.26	88^a ^± 1.73	a
	4h	pH	4.63^a ^± 0.01	4.65^a ^± 0.03	4.5^b ^± 0.01	4.49^b ^± 0.02	a
		Acidity D	80.66^c ^± 1.52	90.66^b ^± 1.52	91.66^b ^± 5.68	99^a ^± 3.60	a
Postacidification	7day	pH	4.19^a ^± 0.03	4.13^b ^± 0.0	4.09^b ^± 0.01	4.09^b ^± 0.01	a
		Acidity D	87.33 ± 5.77	92.66 ± 11.01	100.83 ± 3.32	99.66 ± 3.51	NS
	14day	pH	4.13 ± 0.03	4.15 ± 0.01	4.1 ± 0.01	4.11 ± 0.03	NS
		Acidity D	89.33 ± 6.65	96.66 ± 3.51	99.26 ± 4.19	99.66 ± 8.62	NS
	21day	pH	4.11 ± 0.03	4.04 ± 0.06	4.02 ± 0.01	4.02 ± 0.01	NS
		Acidity D	89.66 ± 7.09	98 ± 8.71	99.1 ± 7.53	100 ± 4.58	NS

The results are expressed as mean standard error followed by NS: not significant (*P* > 0.05) of pectin addition; a, b, c: the homogenous groups after means comparing.

a Highly significant (*P* < 0.01) of pectin addition;

During the period of fermentation, a highly significant decrease (*P* < 0.01) of pH of yogurt added with pectin was recorded, with mean values of 5.09 at 2 h and 4.57 after 4 h of fermentation. Furthermore, the evaluation of acidity during that same period was marked by a highly significant increase (*P* < 0.01) on average of 70.29°D at 2 h to reach 90.50°D after 4 h. During the postacidification period, the decrease in pH was slow and progressive giving mean values of 4.13, 4.12, and 4.05 at 7th, 14th and 21st day of storage at 4°C, respectively. In addition, during this phase, a progressive increase in lactic acid of experimental yogurt was recorded from 95.12 to 96.23 and 96.69°D on average after 7, 14, and 21 days of storage at 4°C, respectively.

According to Luquet (1990), lactic strains have the ability to ferment lactose into lactic acid, with an increase of acidity and a decrease in pH of yohurt. Sokolinska et al. (2004) indicated that, the pH values of milk under processing, from the time it was inoculated with bacterial cultures to the time the yogurt was manufactured, decreased from 6.70 to 4.34. It was demonstrated that during the 21st day period of storage, the pH values decreased to 4.11. *Streptococcus thermophilus* and *Lactobacillus bulgaricus* live in symbiosis and there exists a synergy between the two bacteria which relates to a mutual stimulation. This stimulation relates namely to the growth, acidification, and the production of aromatic compounds.

*Streptococcus thermophilus* is stimulated by the contribution of amino acids and small peptides coming from the proteolytic activity of *Lactobacillus bulgaricus*. The stimulation of *Lactobacillus bulgaricus* is allotted to formic acid, pyruvic acid, and carbon dioxide produced by *Streptococcus thermophilus*. Both microbial species are homofermentative bacteria, which produce lactic acid starting from lactose milk. The production of lactic acid results in a lowering of pH. At the approach isoelectric pH (pHi 4.6), the casein micelles lose their steric stability, thus causing their flocculation, they precipitate and form a coagulum (Loveday et al. 2013).

During the period of fermentation and the first week of postacidification, a relation inversely proportional (*P* < 0.01) was established between the pH values of the experimental yogurt and pectin doses. Furthermore, during the period of fermentation, it appeared that the increase in lactic acid was proportional with the increase in the addition of pectin to 0, 0.1, 0.3, and 0.6% (*P* < 0.01). During the period of postacidification, the addition of pectin had an insignificant effect on the evaluation of lactic acid of yogurt. Kumar and Mishra (2004) found that lactic acidity of yogurt increased with the increasing level of addition of pectin to 0.2, 0.4, and 0.6%.

### Evaluation of viscosity

The viscosity of yogurt supplemented with pectin was characterized by a clear increase during the period of fermentation and postacidification (Table 2). Similar results were obtained by Guzel‐Seydim et al. (2005). According to Girard and Lequart (2007), specific germs of yogurt, particularly, *Streptococcus thermophilus* produce an exopolysaccharide during the lactic fermentation, capable of binding to the casein of milk which confer a viscosity and a particular rheological quality to the finished product. Guzel‐Seydim et al. (2005) found that the viscosity of the fermented milk prepared by bacteria‐producing exopolysaccharide is often much higher than those prepared by bacteria incapable of producing them.

**Table 2:** Evolution of viscosity (Pas) of yogurt added with pectin

Periods		Pectin added doses (%)				Effect of pectin Incorporation
		0%	0.1%	0.3%	0.6%	
Fermentation	2h	3.59^b ^± 0.58	5.58^b ^± 2.11	12.76^a ^± 1.37	12.93^a ^± 1.96	a
	4h	16.36^b ^± 1.39	19.55^b ^± 5.29	28.66^a ^± 7.26	30.68^a ^± 5.24	b
Postacidification	7j	30.98^b ^± 0.67	30.11^b ^± 5.13	38.64^a ^± 1.95	42.17^a ^± 1.48	a
	14j	28.92^c ^± 4.52	35.98^b ^± 3.79	44.42^a ^± 2.34	47.56^a ^± 1.84	a
	21j	33.05^b ^± 1.81	40.18^a ^± 1.55	42.35^a ^± 2.68	45.35^a ^± 4.01	a

Results are expressed as mean standard error followed;

a Highly significant (*P* < 0.01) of pectin addition.

b Significant (*P* < 0.05) of pectin addition; a, b, c: the homogenous groups after means comparing.

The increase in the rate of incorporation of pectin in fermented milks of 0, 0.1, 0.3, and 0.6% was accompanied by a significant increase (*P* < 0.01) of 3.59, 5.58, 12.76, and 12.93 Pas at 2 h at 33.05, 40.18, 42.35, and 45.35 Pas after 21 days of storage at 4°C. The same results were obtained by Jensen et al. (2010) who found that the increase in the pectin concentration from 0.2 to 0.5% leads to the increase in the viscosity of the acidified milk.

Pectin consists essentially of galacturonic acid residues linked by bonds *α* (1→4) partially acetylated or esterified by methyl groups which confer its gelling, thickening, and stabilizing properties. In addition, it has a great water holding (Fishman et al. 2000). These results are explained by the fact that the pectin can form a three‐dimensional network capable of complexing the milk components while absorbing maximum water of the medium resulting in an increase in viscosity of the experimental yogurts (Dickinsom et al. 1998). Pectin is absorbed on the surface of the casein micelles, which form stable aggregates (Maroziene and Kruif 2000; Tuinier et al.^2002^; Kiani et al. 2010).

### Specific germs yogurt

**Figure 1:**
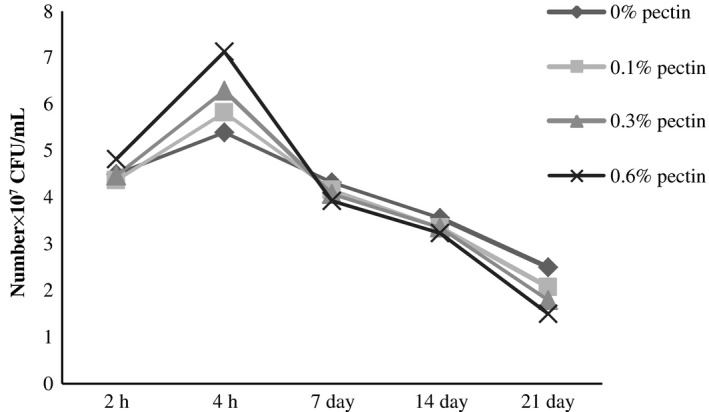
Evaluation of the number of *Streptococcus thermophilus* (CFU/mL) of yogurt added with pectin.

**Figure 2:**
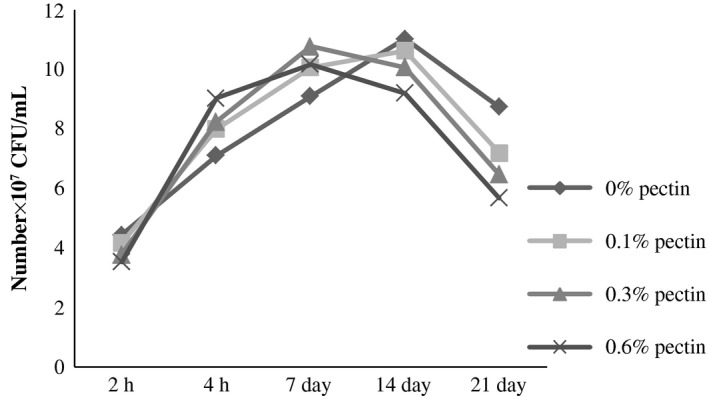
Evaluation of the number of *Lactobacillus bulgaricus* (CFU/mL) of yogurt added with pectin.

The evaluation of the number of *Streptococcus thermophilus* and *Lactobacillus bulgaricus* of yogurt added with pectin was characterized by a continuous increase in the fermentation phase. During the postacidification, the average number of *Streptococcus thermophilus* in the experimental samples was decreased at 4.12 × 10^7^–1.96 × 10^7^ CFU/mL from the beginning to the end of the period of storage (Fig. 1). However, the increase in *Lactobacillus bulgaricus* continued until the 14th day of storage (10.23 × 10^7 ^UFC/mL) and declined thereafter to 7.02 × 10^7^ UFC/mL at 21 days (Fig. 2). It is well established that the *Streptococcus thermophilus* ensure the starting of lactic fermentation of yogurt, and their growth is stimulated by amino acids released because of the proteolytic activity of *Lactobacillus bulgaricus* coming from milk casein. This was translated during the first phase of fermentation by a higher number of *Streptococcus thermophilus*. Then, there is an inhibiting effect of lactic acid which is exerted on *Streptococcus thermophilus* and leads to decrease in their number (Jeantet et al. 2008). In addition, generally, the number of these two strains appeared proportional to the rate of pectin incorporated in fermented milks. A simulative effect of pectin was observed, in particular, in the growth of *Lactobacillus bulgaricus* during the postacidification period. It was through the use of pectin as a carbon source by the specific strains of yogurt (Pak et al. 2013). A clear proportional rise of *Lactobacillus bulgaricus* was observed with the increase of pectin concentrations in yogurt. Kumar and Mishra (2004) found that the rate of incorporation of the pectin in the yogurt has a significant effect on the growth of both strains: *Streptococcus thermophilus* and *Lactobacillus bulgaricus*.

### Sensory analysis

The sensory quality of yogurt was significantly improved with the increase of pectin incorporation rate.

**Figure 3:**
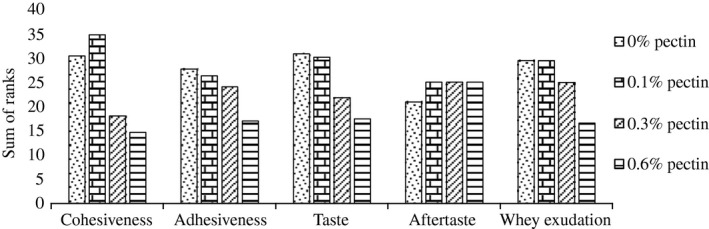
Evaluation of sensory properties of yogurt added with pectin during 21 days of cold storage at 4°C.

Indeed, during the postacidification period, the panelists qualified the experimental yogurt added with 0.6% of pectin having better values of cohesiveness (14.62 sum of ranks) than those prepared with 0, 0.1, and 0.3% of pectin (30.37, 34.75, and 18 sum of ranks, respectively) (Fig. 3). During 21 days of the storage period, adhesiveness tends to increase with the increasing dose of pectin incorporated in yogurt. Similar results were obtained by Kumar and Mishra (2004) who found an improvement of the adhesiveness and the cohesiveness of experimental yogurt with the addition of the pectin rate.

The improvement of the taste of yogurt was proportional with the increase of pectin addition rate. The best taste (17.37 sum of ranks) was obtained with a rate of 0.6% of incorporated pectin. Moreover, the panelists evaluated the controlled fermented milks (0% pectin) as very pleasant aftertaste. The acidity revealed when tasting yogurt is due to a lactic fermentation of milk lactose by the two strains *Lactobacillus bulgaricus* and *Streptococcus thermophilus* (Bourgeois and Larpent 1989). The acetaldehyde formed during lactic acid fermentation is the main constituent of the specific yogurt flavor (Sahan et al. 2008). The interaction between the milk proteins and pectin leads to unfolding of the protein which makes the hydrophobic groups accessible. These groups provide additional sites of fixing of volatile compounds (Mao et al. 2014). These cause a reduction in volatility of the aroma compounds, so a better flavor of yogurt supplemented with pectin. In addition, the increase of the viscosity can influence the mobility of the flavor compounds in the matrix (Mao et al. 2014) which can decrease their release to the gas phase and improve the olfactory perception.

Whey exudation is inversely proportional with the increase of pectin rates incorporated in the yogurt (*P* < 0.01). Similar results were reported by Everett et al. (2005), who found that the addition of pectin to the yogurt improves whey exudation due to the absorption of pectin on the surface of the milk casein micelles, which consequently increases the water‐holding capacity of fermented milk.

## Conclusion

Pectin incorporated in rate of 0.6% significantly improved the rheological quality of the yogurt, in particular: viscosity, adhesiveness and cohesiveness. The addition of pectin also seems to prevent the exudation of whey during the conservation. A better proliferation of *Streptococcus thermophilus* and *Lactobacillus bulgaricus* was observed during the period of fermentation.

## Conflict of Interest

None declared.

[Correction added on 21 October 2016, after first online publication: Reference “Zanelle, K., and O. P. Taranto. 2015” has been changed to “Zanella, K., and O. P. Taranto. 2015”.]
